# Identification and Validation of a New Source of Low Grain Cadmium Accumulation in Durum Wheat

**DOI:** 10.1534/g3.117.300370

**Published:** 2018-01-19

**Authors:** Atena Oladzad-Abbasabadi, Ajay Kumar, Seyed Pirseyedi, Evan Salsman, Marina Dobrydina, Roshan Sharma Poudel, Wesam A. AbuHammad, Shiaoman Chao, Justin D. Faris, Elias M. Elias

**Affiliations:** * Department of Plant Sciences, North Dakota State University, Fargo, North Dakota 58108; † Arizona Plant Breeders Inc., Casa Grande, Arizona 85122; ‡ Department of Plant Pathology, North Dakota State University, Fargo, North Dakota 58108; § United States Department of Agriculture-Agricultural Research Service, Red River Valley Agricultural Research Center, Cereal Crops Research Unit, Fargo, North Dakota 58102

**Keywords:** QTL mapping, cadmium (Cd), durum wheat, allelism test, single nucleotide polymorphism

## Abstract

Cadmium (Cd) is a heavy metal that has no known biological function and is toxic for many living organisms. The maximum level of Cd concentration allowed in the international market for wheat grain is 0.2 mg kg^−1^. Because phenotyping for Cd uptake is expensive and time consuming, molecular markers associated with genes conferring low Cd uptake would expedite selection and lead to the development of durum cultivars with reduced Cd concentrations. Here, we identified single nucleotide polymorphisms (SNPs) associated with a novel low Cd uptake locus in the durum experimental line D041735, which has hexaploid common wheat in its pedigree. Genetic analysis revealed a single major QTL for Cd uptake on chromosome arm 5BL within a 0.3 cM interval flanked by SNP markers. Analysis of the intervening sequence revealed a gene with homology to an aluminum-induced protein as a candidate gene. Validation and allelism tests revealed that the low Cd uptake gene identified in this study is different from the closely linked *Cdu1-B* gene, which also resides on 5BL. This study therefore showed that the durum experimental line D041735 contains a novel low Cd uptake gene that was likely acquired from hexaploid wheat.

Cadmium (Cd) is a heavy metal present in the environment (soil, air, and water), and has no known biological function in plants. High Cd concentrations are not toxic to plants (Grant *et al.* 1998). However, translocation of Cd from soils to edible crops and then to humans and animals through direct or indirect food sources, has toxic effects in many living organisms. High Cd intake is carcinogenic for humans and can cause serious damage to the kidneys (Grant *et al.* 1998; Olsson *et al.* 2005; Menke *et al.* 2009; Nishijo *et al.* 2006).

Durum wheat (*Triticum durum* L. var. *durum* Desf.) is used to make pasta and other semolina-based products, and is therefore a major food source for humans. Although most plant species accumulate Cd in the roots, durum has a tendency to accumulate high amounts of Cd in the grains. Grain harvested in North Dakota has been reported to have Cd levels ranging from 0.025 to 0.359 mg kg^−1^ (Knox *et al.* 2009; Wu *et al.* 2002). Given the importance of durum wheat both economically (National Agricultural Statistics Service 2015) and as a major food source, the development of varieties with low Cd levels is an important objective of durum wheat breeding programs. Unfortunately, chemical analysis for identifying low grain Cd phenotypes is costly and time consuming. The use of genomic tools could be a more practical and efficient approach for selecting low Cd lines in durum wheat.

In durum wheat, Penner *et al.* (1995) mapped a RAPD (Random Amplification Polymorphic DNA) marker (OPC 20) linked 5 cM from the Cd uptake gene *Cdu-B1*, which was later located on chromosome arm 5BL (Knox *et al.* 2009). Subsequent saturation (Wiebe *et al.* 2010) and fine mapping (Wiebe 2012) experiments led to the identification of markers that cosegregated with *Cdu-B1*, including the CAPs (Cleaved Amplified Polymorphic Sequence) marker *Xusw47*, and delineated the candidate region to a 0.14 cM interval containing the candidate Cd uptake gene *TdHMA3-B1*. Pozniak *et al.* (2012) found five DArT (Diversity Arrays Technology) markers linked with phenotypic grain Cd concentration. Three of these markers, including *wPt-1733*, *wPt-2453*, and *wPt-9300*, explained similar proportions of the phenotypic variance as the *Xusw47* marker.

Recently, SNP arrays have become powerful tools for identifying marker-trait associations. Advances in high-throughput sequencing led to the development of the iSelect SNP array containing nearly 90,000 wheat SNP markers, which provides an opportunity to genetically dissect loci controlling target phenotypes at a high resolution (Wang *et al.* 2014). Tightly linked user-friendly markers developed from these SNPs are more effective in molecular wheat breeding due to their high density, speed and cost effectiveness. Detecting SNP markers for important traits such as Cd uptake and subsequently developing user-friendly markers from these SNPs can expedite selection progress in durum breeding programs.

SNP markers linked to the Cd accumulation locus in some North Dakota durum cultivars were recently reported by AbuHammad *et al.* (2016). In that study, the SNP marker *IWA1775* was found tightly associated with grain Cd content. The RAPD marker *OPC20* and the KASPar genotyping assay developed from the SNP marker *IWA1775* were able to separate low Cd content lines from high Cd content lines (AbuHammad *et al.* 2016). However, the populations used by AbuHammad *et al.* (2016) have parental backgrounds from two main sources of low grain Cd; the Canadian cultivar Strongfield (Clarke *et al.* 2005), and the Syrian cultivar Hurrani. Strongfield is adapted to the western part of the North Dakota durum-growing region, whereas Hurrani is unadapted for this region.

An experimental line designated as D041735 was recently developed by the durum wheat breeding program at NDSU (North Dakota State University). The purpose of developing this line was to integrate *Fusarium* head blight (FHB) (caused by *Fusarium graminearium* Schwabe) resistance from the hexaploid wheat cultivar “Sumai 3” into durum wheat. D041735 shows Cd levels even lower than those of Strongfield and Haurani. Neither Strongfield nor Haurani were involved in the pedigree of D041735, suggesting the possibility of a novel gene or allele controlling low grain Cd in this experimental line. Any new source for low Cd uptake could be used to produce high quality durum wheat that meets food safety and exportability requirements. Also, the genetic basis of Cd accumulation in durum wheat is still not well defined. Therefore, the objectives of this study were to (1) dissect the genetic component(s) controlling low grain Cd content in the durum experimental line D041735 using a recombinant inbred line (RIL) mapping population and a high density SNP-based map; (2) identify and validate SNP marker(s) tightly linked to loci controlling Cd uptake; and (3) determine if the gene(s) controlling Cd uptake in D041735 are different from those controlling Cd uptake in Strongfield and Haurani.

## Materials and Methods

### Plant materials

Two experiments were conducted for this study. For the first experiment, a RIL mapping population was developed from a cross between the low Cd uptake line D041735 and the high Cd uptake cultivar “Divide” (Elias and Manthey 2007). Divide is a high yielding durum wheat cultivar with excellent quality and a moderate level of FHB resistance released by NDSU. D041735 is a low Cd uptake durum wheat experimental line developed by the durum wheat breeding program at NDSU. This line was developed using hexaploid wheat Sumai 3 as one of the parents, (Lebsock/Sumai 3//Lebsock/3/Lebsock/4/Lebsock). The RIL population was developed using the single-seed descent method. A total of 190 RILs (F_4:6_), two parents and four checks that varied in their Cd uptake levels were planted at two locations in a 14 × 14 lattice design with two replications. The locations were Langdon (48°45′42″N 98°22′18″W) and Prosper (46°96′30″N, 97°01′98″W) in North Dakota. The soil type in Langdon is Svea and Barns, and it is Perella and Bearden in Prosper. The checks used in this study were low Cd uptake genotypes Strongfield and “CDC-Verona” (Pozniak *et al.* 2009), and high Cd uptake genotypes Carpio (Elias *et al.* 2015) and “Joppa” (Elias and Manthey 2016).

This experiment also employed 144 genotypes for validation of the identified locus for Cd uptake. These lines included, (1) a population of 50 RILs derived from a cross between the low Cd genotype D041735 and the high Cd cultivar Joppa, and (2) 94 breeding lines developed from 12 different crosses between the low Cd uptake and high Cd uptake genotypes with no genetic background from D041735. The materials were planted in 2014 (50 RILs) and 2013 (94 RILs) in two locations (Williston and Langdon, ND) as a 10 × 10 lattice design with two replications. Phenotypic selections for low Cd were made throughout the development of these lines (Supplemental Material, Table S1).

For the second experiment, an allelism test was performed using the three sources for low Cd uptake in the NDSU durum wheat breeding program including D041735, Haurani (Syrian durum) and Strongfield (Canadian durum). These genotypes were intercrossed in the fall of 2012 to develop three segregating populations for allelism testing. The single-seed descent method was used to develop a RIL population for each cross. In 2014, F_6_ RILs from each of the three populations were planted in a 13 × 13 lattice experiment with two replications, at two locations (Prosper and Langdon, ND). For each of the three populations, a total of 169 individuals including 162 RILs and four low Cd checks (D041735, Haurani, Strongfield, and CD-Verona) and three high Cd checks (Divide, Carpio, and Joppa), were evaluated for grain Cd content. Data collection and analysis were done as described for the previous experiment.

### Phenotyping

To phenotype the grain Cd content, eight spikes of each genotype were randomly harvested using chromium knives. These spikes were threshed using an uncolored metal thresher to prevent any risk of Cd contamination in the samples. Seeds from one spike were kept as a remnant, some of which were later grown in the greenhouse for DNA extraction. The seeds from seven spikes for each genotype were sent to the College of Agriculture and Life Science, Nutrient Analysis Laboratory at Cornell University in Ithaca, New York, to estimate grain Cd concentration. For this purpose, seeds from each genotype were milled and dried in an oven. Then, 0.5 g of the flour of each genotype was dissolved into a solution consisting of nitric and perchloric acids. To break down the flour into its components, the mixture of each sample was placed in a fluorocarbon container and heated to 180 ± 5° for <5.5 min, and remained at the same temperature for another 9.5 min. After the heating process ended, the samples remained in the heater for a minimum of 5 min to cool before removing. Once the containers were sufficiently cooled (near room temperature), each sample was diluted with ∼10% v/v nitric acid up to 20 ml in volume. Finally, a SW-846 method defined by the environmental protection agency (EPA method No. 3050, 3051, and 3052 https://www.epa.gov/sites/production/files/2015-12/documents/3052.pdf, https://www.epa.gov/sites/production/files/2015-12/documents/3051a.pdf, and https://www.epa.gov/sites/production/files/2015-06/documents/epa-3050b.pdf) was used to analyze the concentration of Cd and other elements for each sample.

### Statistical analysis

The phenotypic data were analyzed for each location separately using the Statistical Analysis System SAS 9.3 (v9.3; SAS Institute Inc., Cary, NC). Homogeneity of error variances between the two locations were tested by Levene’s, as well as Brown and Forsythe’s, homogeneity tests. Analysis of the variance across locations was also performed to determine if there were significant genotype by location interactions. In the statistical analysis, RILs (genotypes) were considered fixed effects, while locations, replication within locations, block per replication per RIL, and location per RIL were treated as random effects. Means were scored and separated by Fisher’s Protected least significant differences (LSD) at the 5% level of significance.

### DNA isolation

Seeds from each genotype were grown in the greenhouse. Two inches of leaf tissue were collected for each genotype and used for DNA extraction. The leaf tissues were collected in 96-well plates containing 2.5 mg of silica gel. Samples were then sent to the United States Department of Agriculture-Agricultural Research Service small grains genotyping laboratory in Fargo (ND) for molecular analysis. The DNA was extracted following Pallotta *et al.* (2003). The protocol can be found on the United States Department of Agriculture website: http://wheat.pw.usda.gov/GenotypingLabs/fargo.html.

### Genotyping using Illumina 90k SNP assay

The RIL population developed from D041735 and Divide, the parental genotypes and the checks, were genotyped using the Illumina iSelect 90K wheat SNP arrays (Wang *et al.* 2014) at the United States Department of Agriculture-Agricultural Research Service small grains genotyping laboratory in Fargo (ND). The genotyping data were analyzed using the diploid version of Illumina’s GenomeStudio Software (https://www.illumina.com/techniques/microarrays/array-data-analysis-experimental-design/genomestudio.html). The necessary corrections for each genotype were done manually to make sure all possible errors related to cluster assessment were edited. Moreover, SNPs with a high missing data rate (>20%) and low allele frequency (<0.4) for any of the parental genotypes were removed.

### Genetic linkage map construction

To construct the SNPs linkage map for each chromosome, genotyping data from the GenomeStudio software were exported to an Excel file. Markers were sorted according to the parental genotypes. Initially, all monomorphic markers were deleted. From the final list of polymorphic markers, a total of 7–10 markers per chromosome were selected as anchors for developing linkage maps using MapMaker 3.0 (Lander *et al.* 1987). The markers were selected to represent the length of the chromosome based on a published durum wheat consensus map (Maccaferri *et al.* 2015). MapMaker was used to also generate linkage groups based on a minimum LOD score of 3.0 and a maximum recombination frequency of 50%. The markers within each linkage group were then imported into CartaGene V.1.2.3R (de Givry *et al.* 2005) to estimate the marker order and genetic distances as described elsewhere (Kumar *et al.* 2012a,b, 2016). The Kosambi mapping function (Kosambi 1943) was used to convert recombination frequencies into map distances (centimorgans).

### Quantitative trait loci (QTL) analysis

QTL analysis was performed on individual environmental data as well as on the mean data across environments using the Inclusive Composite Interval Mapping (ICIM) method available in the software QTL IciMapping V 4.1 (Meng *et al.* 2015). Only unique loci were used for each linkage group. Markers for a linkage group were defined by the cumulative distance. Permutation tests (1000 iterations) were conducted to determine the significant LOD threshold values (α _0.01_ and α _0.05_ experiment-wide error).

### KASPar assay development for validation testing

The sequences of two flanking SNPs associated with a major QTL for Cd were used to develop KASPar primers (Kompetitive Allele Specific PCR; LGC Ltd., Teddington, United Kingdom). For the assay, DNA samples were first added to 96-well plates using a matrix 2 × 2 robot. The samples were then dried at 65°. A PCR reaction was prepared from 2 µl water, 2 µl master mix (specific for the Roche Light Cycler ordered from LGC), and 0.055 µl allele specific primer. The reactions were then placed on an ABI GeneAmp PCR machine for 32 cycles, and the plates read on a Roch Light Cycler 480 (Roche Life Sciences). The KASPar markers designed from the SNPs flanking the major QTL were used for the validation test.

### Identification of putative candidate genes

The nucleotide sequence of the markers *IWB47298*, *IWB34332*, and *IWB55063* associated with Cd uptake were used to conduct a BLAST search against the wheat draft genome sequences TGACv1 and the newly available wheat genome reference sequence IWGSC RefSeq v1.0. (http://plants.ensembl.org/Triticum_aestivum/Tools/Blast, Deng *et al.* 2007; https://urgi.versailles.inra.fr/blast_iwgsc/blast.php). The chromosomal coordinates of these SNPs were obtained from the BLAST search results against the wheat reference sequence. The sequences spanning the SNPs were extracted using getfasta command from Bedtools suite (Quinlan and Hall 2010). The extracted sequences were used to predict open reading frames (ORFs) in FGENESH using parameters setting specific for *Triticum aestivum* (Solovyev *et al.* 2006; http://www.softberry.com/berry.phtml?topic=fgenesh&group=programs&subgroup =gfind). Putative functions were assigned to the ORFs by conducting BLAST searches against the NCBI (https://blast.ncbi.nlm.nih.gov/Blast.cgi), IPK (http://webblast.ipk-gatersleben.de/barley_ibsc/), and rice (http://rice.plantbiology.msu.edu/analyses_search_blast.shtml) databases.

### Genotyping for allelism test

The parents and checks from the second experiment were genotyped using two types of markers associated with Cd uptake including KASPar assays, which were developed from the Cd uptake-associated SNP markers identified in experiment 1, and the CAPs marker *Xusw47* previously identified by Wiebe (2012). This CAPs marker was developed from ESM marker *XBF474090*. The recommended PCR annealing and digestion temperatures for this marker are 55 and 37°, respectively. To date, closest marker reported for *Cdu1-B* is *Xusw47*. Therefore, identifying the location of this marker in the genetic map with reference to the major QTL and the SNP markers associated with Cd uptake identified in experiment 1 was of interest. After the polymorphism between the D041735, Haurani, and Strongfield was confirmed by the CAPs and KASPar markers, the DNA of the RIL populations were subjected to PCR amplification using these markers. The DNA for 14 individuals from each population were not available because of germination problems. Therefore, a total of 155 individuals each from the D041735 × Strongfield and Haurrani × D041735 populations as well as Sumai 3 were genotyped. The population derived from the cross between Haurrani and Strongfield was not genotyped due to lack of polymorphism for the Cd-associated markers.

### Data availability

Table S1 (an Excel file) contains detailed descriptions of all materials and results for the validation test. Table S2 (an Excel file) contains all genotyping information and query sequences used in the BLAST search. Table S3 (an Excel file) contains information related to the efficiency of identified markers. Table S4, Table S5, Table S6, and Table S7 (Word documents) contain the phenotypic data of the allelism test for two populations in two locations. Figure S1 includes the linkage map of the durum genome.

## Results and Discussion

### Phenotypic analysis of the divide × D041735 RIL population

D041735 and Divide differed significantly for levels of grain Cd (Table 1). The Cd level among progenies varied from 0.021 to 0.242 mg/kg in Langdon, and from 0.092 to 0.524 mg/kg in Prosper. The continuous variation for Cd content for the RILs in the two environments suggested that Cd uptake was quantitatively controlled. High heritability in both locations (83% in Langdon and 82% in Prosper) agreed with the results of a previous study (Clarke *et al.* 1997), and suggested that genetic components play a larger role in grain Cd uptake than environmental factors. Nineteen RILs in Langdon and 14 RILs in Prosper showed a lower Cd content than D041735, the low Cd parent. Moreover, 50 RILs in Langdon and 37 RILs in Prosper showed Cd levels higher than the high Cd parent Divide. These transgressive phenotypes in the mapping population could be the result of environmental factors, epistatic interactions or both genotypes contributing low Cd alleles at some minor loci. The results of Levene’s homogeneity test differed from the results of Brown and Forsythe’s homogeneity test (data not shown). Therefore, the data were not pooled across locations, and the data from individual environments was analyzed separately instead.

**Table 1:** Mean Cd uptake of parents and checks and the range of the mapping population

D041735 × Divide (RIL Population)		
Parents	Cd Content (mg/kg)	
	Langdon	Prosper
D041735	0.034	0.140
Divide	0.113	0.387
Checks		
Strongfield	0.053	0.138
CD-Veronica	0.056	0.250
Joppa	0.128	0.286
Carpio	0.124	0.320
The range of the data		
Minimum	0.021	0.092
Maximum	0.242	0.524
LSD	0.047	0.142

### Linkage mapping

After filtering the genotypic data, a total of 3973 polymorphic SNP markers were selected for constructing a linkage map. Out of those markers, a total of 3923 SNPs were assembled into 28 linkage groups, representing portions of each of the 14 durum wheat chromosomes. This was not surprising considering the fact that both Divide and D041735 are products of the same breeding program. The two lines share some of their pedigrees and thus we would expect large regions to be essentially void of polymorphism resulting in rather large gaps between linkage groups.

The total map length was 2137.5 cM. The 3923 markers represented 849 unique loci, with an average distance of 2.52 cM between two loci (Figure S1 and Table S2). This study is the first to report the use of a high-density 90K SNPs assay for dissection of Cd uptake in wheat, suggesting a more robust QTL detection approach compared to previous QTL mapping studies. A recent study on the genetic dissection of Cd in durum wheat used 9K iSelect SNP array and 255 SSRs to assign a total of 330 markers on the whole durum genome (AbuHammad *et al.* 2016). The framework linkage map in this study assigned more SNPs onto the B-genome compared to the A-genome. This coincides with the findings of previous studies, which found more diversity in the B-genome of wheat than the A-genome (Petersen *et al.* 2006; Feldman and Levy 2012; Panchy *et al.* 2006).

### QTL analysis

**Figure 1:**
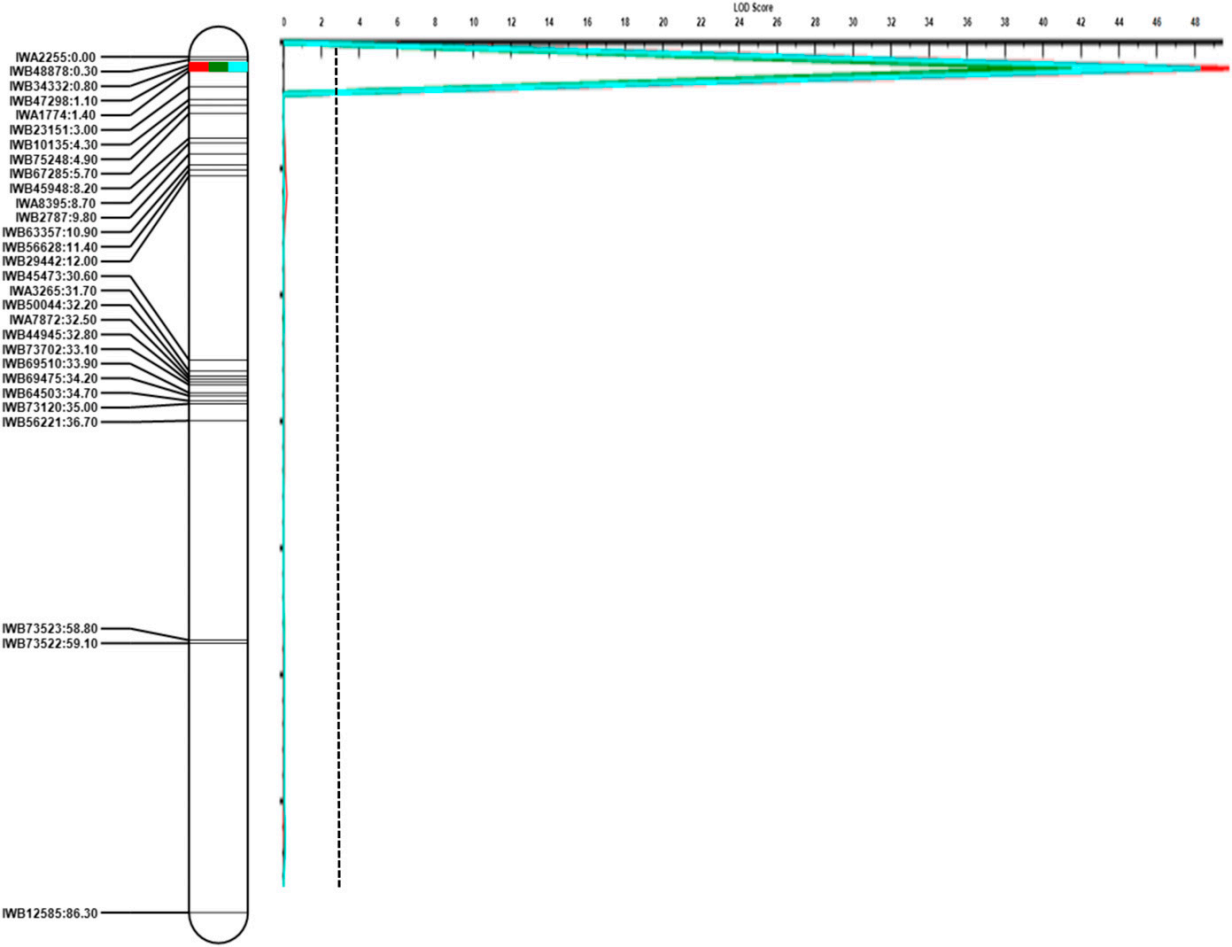
A major QTL for Cd grain uptake detected in the D041735 × Divide durum wheat population across two locations (Langdon and Prosper, ND, indicated with the green and red curves, respectively) and mean (the blue curve) on chromosome 5B. The significant LOD threshold was estimated at 2.38, 1.88, and 2.07 for Prosper, Langdon, and mean, respectively. The largest LOD threshold is shown by the dashed line. The figure includes only nonredundant markers.

QTL mapping revealed one major QTL (designated *QCdu.ndsu-5B*) associated with Cd uptake in both field environments. The SNPs *IWB34332* (proximal) and *IWB47298* (distal) on 5B with 0.3 cM distance between them tightly flanked this QTL, which had a LOD score of ∼50. The proximal flanking marker also cosegregated with *IWB55063*, but the distal marker was a unique locus (Figure 1 and Table 2). The Divide allele at the major locus added an average of 0.06 mg/kg of Cd into the seeds (0.031 mg/kg in Langdon, and 0.086 mg/kg in Prosper). *QCdu.ndsu-5B* explained ∼70.6% of the phenotypic variation in seed Cd content.

**Table 2:** Summary of the detected major and minor QTL for grain Cd uptake in the RIL population derived from D041735 × Divide at two locations (Langdon and Prosper, ND) and the mean

Location	QTL	Marker Interval	Marker Position (Cumulative Distance cM)	Divide × D041735		
				LOD	*R*^2^	Add
Langdon	*QCdu.ndsu-5B*	*IWB34332/IWB55063-IWB47298*	0.8–1.1	49.46	70.6	0.031
Prosper	*QCdu.ndsu-5B*	*IWB34332/IWB55063- IWB47298*	0.8–1.1	41.15	64.0	0.086
Mean	*QCdu.ndsu-5B*	*IWB34332/IWB55063- IWB47298*	0.8–1.1	47.96	69.5	0.059
Langdon	*QCdu.ndsu-4B*	*IWB57047- IWB29236*	85.3–86.1	2.29	1.6	0.004
Prosper	*QCdu.ndsu-4A*	*IWB6376- IWB29840*	0.9–5.3	2.12	2.1	0.014

**Figure 2:**
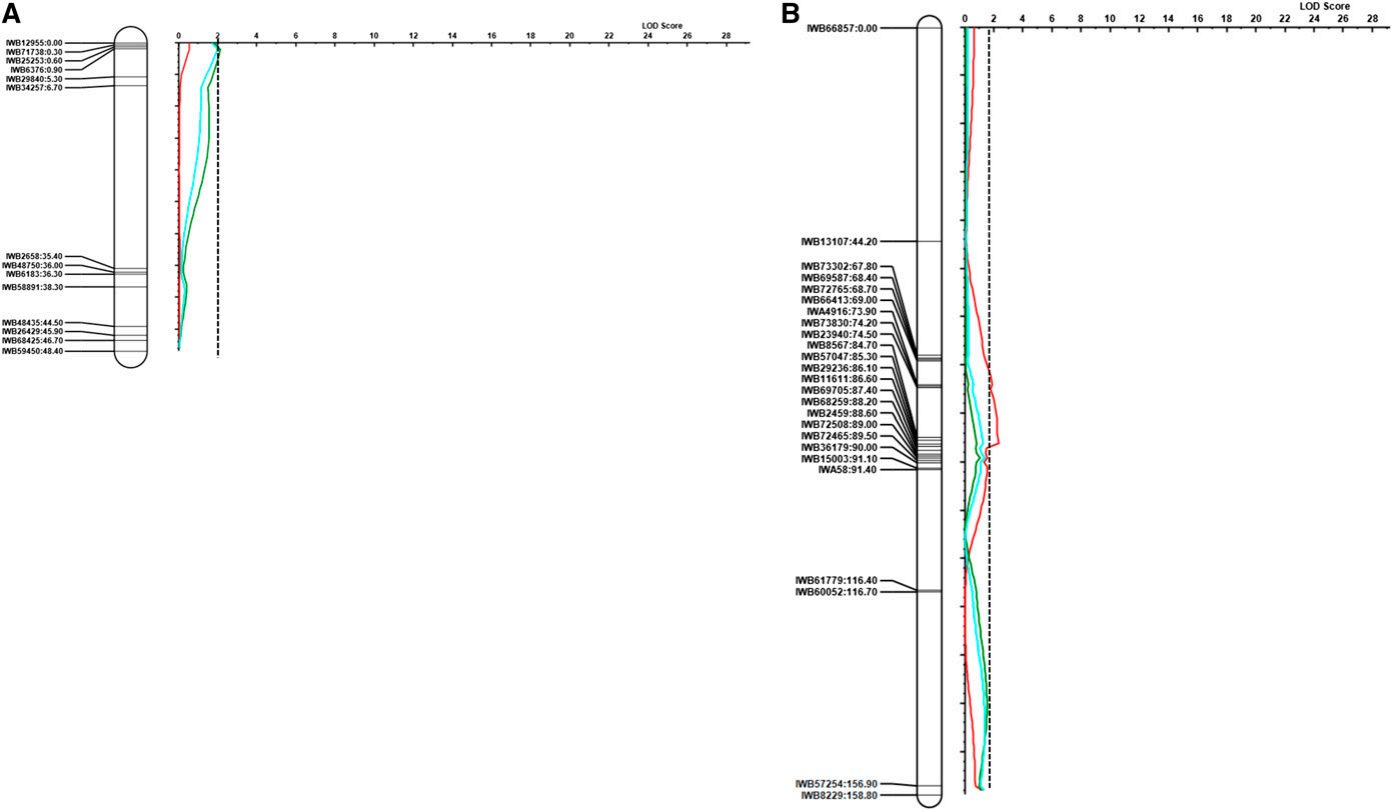
Minor QTL detected in the D041735× Divide population for grain Cd Content on Chromosome 4A (A) and Chromosome 4B (B). The red curve indicates the minor QTL detected in Langdon. The green curve indicates the minor QTL detected in Prosper. The blue curve indicates the QTL detected using mean data. The significant LOD threshold was estimated 2.38, 1.88, and 2.07 for Prosper, Langdon, and mean, respectively. The largest LOD threshold is shown by the dashed line. The figure includes only non-redundant markers.

Previous studies also indicated a major QTL for Cd uptake on chromosome 5B (Knox *et al.* 2009; Wiebe *et al.* 2010; AbuHammad *et al.* 2016). AbuHammad *et al.* (2016) placed the major Cd QTL among markers that explained 54.3% of variation in grain Cd accumulation. In addition to *QCdu.ndsu-5B*, two QTL with a minor effect were also identified, one each on chromosomes 4B and 4A (Figure 2 and Table 2) designated *QCdu.ndsu-4B* and *QCdu.ndsu-4A*, respectively. These minor QTL were specific to each location. *QCdu.ndsu-4B* showed an additive effect of only 0.0047 mg/kg Cd content in the Langdon location, whereas *QCdu.ndsu-4A* had an additive effect of 0.015 mg/kg Cd content in the Prosper location. Minor QTL for Cd have been reported previously on chromosomes 2B and 5B (AbuHammad *et al.* 2016; Wiebe *et al.* 2010). As Uemoto *et al.* (2015) stated, detecting minor QTL and digenic interaction effects are important for estimating heritability and increasing predictive accuracy in breeding programs.

Knowledge about the existence of such effects might open a new window toward a better understanding of the relative differences in the range of Cd content phenotypes in different environments. For example, individuals in the Prosper location consistently showed a higher Cd uptake compared to those at Langdon. Some possible environmental factors in these two locations might have an effect on up- or downregulation of some minor QTL associated with Cd uptake. Lower heritability in the Prosper location also indicates a slight genotype by environment (G × E) interaction. Therefore, using statistical tools like QTLNetwork (Yang *et al.* 2008) can be useful to detect the possible gene network and its interactions with the environment for any trait.

### Effectiveness of selection for the major Cd QTL

The two marker loci flanking *QCdu.ndsu-5B* (*IWB47298* and *IWB34332*/*IWB55063*) each segregated in a 1:1 ratio. To estimate the efficiency of selection for the Cd uptake gene, a table was generated based on the genotypic and phenotypic data of these two flanking markers for the RIL population (Table S3). As shown in the table, only 13 out of 184 lines were misclassified according to their phenotypes, meaning that 93% of the RILs were successfully divided into either low or high Cd classes based on the different alleles for these two markers. More importantly, only six individuals with a high Cd level fell into the low Cd category, which means that 96.5% of the lines were accurately selected based on desirable marker alleles.

### Identification of putative candidate gene for the major Cd uptake locus

The BLAST search using the contextual nucleotide sequence of *IWB47298* as a query against wheat draft genome TGACv1 revealed a match with the gene *TRIAE_CS42_5BL_TGACv1_405827_ AA1335930.1*. The deduced amino acid sequence of this gene was used to identify a homologous barley sequence using a BLAST search of the IPK barley database (Deng *et al.* 2007). The search revealed that the wheat sequence had 100% identity to the barley gene *HORVU5Hr1G094370*, which was predicted to be an aluminum-induced protein (AIP) with YGL and LRDR motifs. Similar BLAST searches were conducted using the genes predicted from the sequences between SNPs *IWB47298* and *IWB34332*, and one of the predicted genes showed 100% identity to the same AIP.

To date, the functions of AIP families in plants have not been clearly described. However, in hexaploid wheat, a domain present in AIP called Wali 7 (wheat aluminum induced 7) was isolated from the root with no known function for the encoded protein (Snowden and Gardner 1993). It was recently shown that the transcription of AIP containing the Wali 7 domain increased when a plant was exposed to aluminum, Cd, and copper compared to a control treatment (Jang *et al.* 2014). Expression occurs in the plasma membrane, which was expected because it is where the plant is able to protect itself against heavy metal transportation into the vital organs (Singh *et al.* 2015). Moreover, a proteomics profiling analysis showed that the Wali 7 domain is an important osmotic resistance protein in hexaploid wheat (Ma *et al.* 2016). These observations suggest a possible involvement of this candidate gene in the common mechanism of plant response to the heavy metal stress condition in this study. This is the first study to report this gene as a candidate for a Cd uptake locus.

### KASPar assay development for the Cd uptake QTL

For a user friendly and cost effective application of Cd associated markers in breeding programs, the markers flanking *QCdu.ndsu-5B* for Cd uptake were converted to KASPar markers (Table 3). The designed KASPar assays separated the high accumulator parent (Divide) from the low accumulator parent (D041735) in the initial test by assigning SNP allele T to the low Cd parent and allele C to the high Cd parent for *IWB47298*. For the second flanking marker, *IWB34332*, KASPar assays assigned SNP allele C for the low Cd parent and allele T for the high Cd allele.

**Table 3:** Allele assignment to the genotypes of the high and low Cd grain level parents

Marker Name	High Cd Allele from Divide	Low Cd Allele from D041735
*IWB47298*	C	T
*IWB34332*	T	C

The results of our first experiment suggested that selection based on the two markers flanking *QCdu.ndsu-5B* will be highly effective for successfully separating low Cd lines from high Cd uptake lines in the population. Because the estimation of Cd in grain samples is very expensive ($18–$25 per sample) and time consuming, the markers identified in this research should lead to significant savings in terms of cost and time by allowing selection of low Cd lines in early generations.

### Validation test

In the D041735 × Joppa RIL population, the phenotypic analysis showed Cd level diversity in both locations. Screening those RILs with KASP markers for *IWB47298* and *IWB34332* showed that most low and high Cd lines in the D041735 × Joppa population had the same segregating marker alleles as were observed in the D041735 × Divide population (Table S1). As the table shows the markers successfully classified 86% of the genotypes into the expected Cd level; however, three high Cd genotypes fell into the low Cd category which means the risk for using the marker for this population was 6%. This validated the utility of the markers in a different population developed using D041735.

On the contrary, unexpected results were observed when we screened breeding lines developed from the other low Cd genotypes, using the same markers. Phenotypically, about half of the lines showed low Cd phenotypes. However, when screening with *IWB47298* and *IWB34332*, all 94 lines showed the markers alleles that were associated with high Cd in the D041735 × Divide population. Similarly, the two other low Cd durum sources, Strongfield and Huarani, had the same allele as the high Cd checks Divide and Joppa (Table S1). Therefore, we were able to validate the Cd-associated markers in populations developed using the low Cd source D041735, but the same markers could not predict Cd in lines derived from other low Cd durum sources. This suggested that the hexaploid wheat-derived low Cd source D041735 might contain different low Cd uptake gene(s) compared to the low Cd sources Haurani, Strongfield, and Transend. Therefore, we conducted further experiments to test this hypothesis.

### Allelism test

**Figure 3 (A–D):**
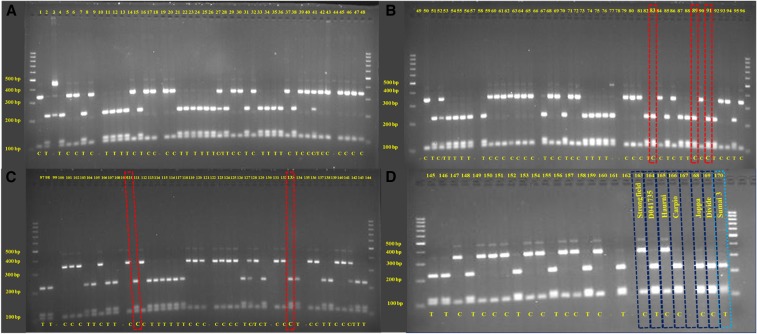
Genotyping results of *Xusw47* (banding patterns; 220 bp: high Cd, 350 bp: low Cd) and designed KASPar of *IWB47298* (letters at the bottom; T: low Cd, C: High Cd) for RILs derived from the cross between D041735 and Strongfield. In (B and C) red rectangles indicate lines with high Cd content both phenotypically and genotypically. In (D) dark blue rectangles indicate checks and light blue rectangles indicates Sumai3 (hexaploid wheat).

*Xusw47* was polymorphic between high Cd uptake genotypes Divide, Carpio, and Joppa, and low Cd uptake genotypes Haurani and Strongfield, and showed expected alleles (Figure 3). However, when amplified with *Xusw47*, D041735 showed the same PCR amplicon (220 bp) as the high Cd uptake genotypes Divide, Carpio, and Joppa, as opposed to the 350 bp amplicon observed in the other low Cd genotypes Strongfield and Haurani. In addition, when genotyped with *IWB47298* (KASP), Strongfield and Haurani had the high Cd uptake SNP allele (C) present in Divide, Carpio, and Joppa.

**Figure 4:**
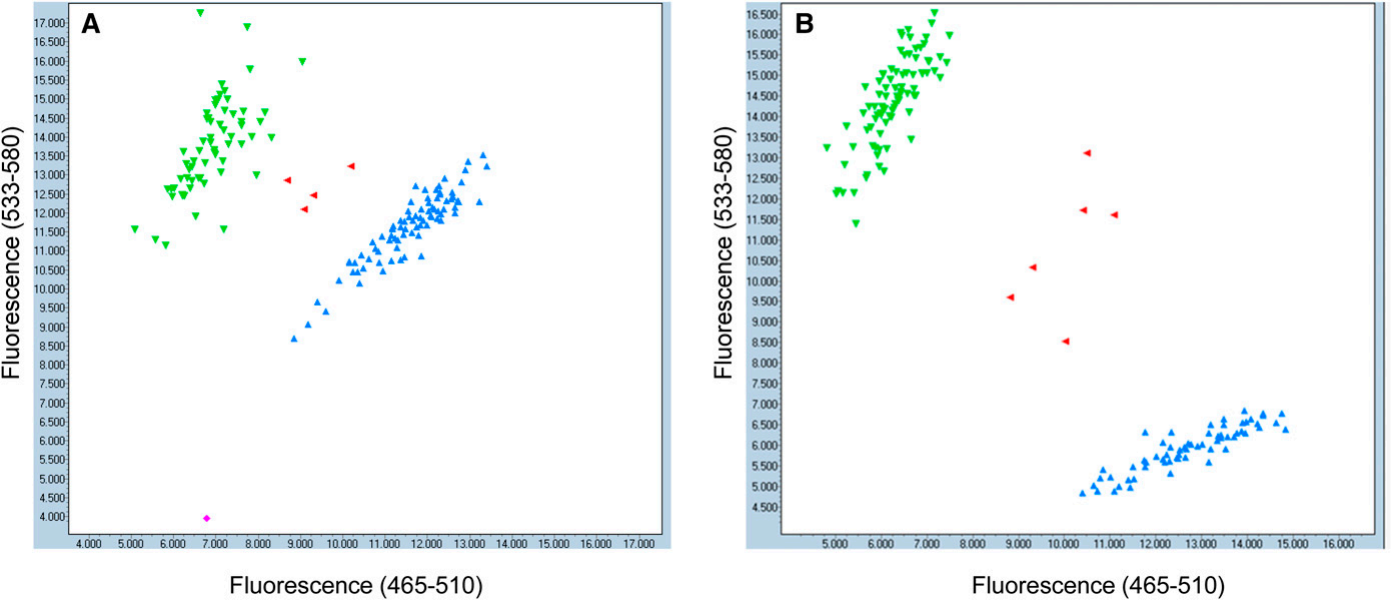
Polymorphism results of running KASPar assay *IWB47298* (A) and *IWB34332* (B) on the RIL population derived from two sources of low Cd; D041735 × Strongfield. Green and blue colors are individuals with low and high Cd alleles respectively. Red color represents individuals with no call.

As both *Xusw47* and *IWB47298* were polymorphic between D041735 (low Cd uptake) and Strongfield (low Cd uptake), they were used to genotype an RIL population developed from these two low Cd uptake parents. The results showed that 73 RILs had Strongfield alleles and 75 RILs had D041735 alleles (Figure 3 and Figure 4). Very few RILs were heterozygous as expected in the F_6_ generation of the population. The RIL population had a mean of 0.35 and 0.084 mg/kg grain Cd level in Prosper and Langdon, respectively (Table S4). The range for grain Cd was from 0.14 to 0.51 mg/kg in Prosper, and from 0.014 to 0.044 mg/kg in Langdon. Langdon always shows “relatively” lower Cd phenotype compare to Prosper for the same genotypes Phenotypic analysis of the Prosper data using LSD between the means of progenies and the parents showed that five individuals were significantly different from both of the low Cd parents and displayed a high Cd phenotype. Therefore, the phenotypic data categorized 143 individuals with a low Cd uptake level and five individuals with a high Cd uptake level. Similarly, the phenotypic analysis of the Langdon data categorized these five lines as high Cd uptake lines relatively to parents (Table S5). The genotypic data based on *Xusw47*(CAPs) and *IWB47298* (KASP) showed that these five individuals had the same alleles as the high Cd check, *i.e.*, 220 bp amplicon for *Xusw47* and SNP allele “C” for *IWB47298*. Comparatively, in 143 low Cd lines, we observed that almost half of the lines had the Strongfield low Cd allele, and the remaining half of the lines had the D041735 low Cd allele. The five high Cd lines had the high uptake alleles at both marker loci.

It is important to emphasize that, in the 143 low Cd lines, lines that displayed the low Cd allele for *Xusw47* had the high Cd allele for *IWB47298*, and alternately, the lines that had the low Cd allele for *IWB47298* had the high Cd allele for *Xusw47*. This clearly indicates that there are different genes governing Cd uptake in D041735 and Strongfield, and the genes are closely linked.

Because Haurani and Strongfield showed the same allelic pattern for Cd associated markers (*Xusw47* and *IWB47298*), we developed a population from the cross between D041735 and Haurani for additional allelism testing and validation of the results observed from analysis of the Strongfield × D041735 population. The D041735 × Haurani RIL population showed the same allelic patterns for the markers *Xusw47* and *IWB47298* as was observed for the D041735 × Strongfield population. Analysis of this population at the Langdon and Prosper locations indicated that six individuals were significantly different from both of the low Cd parents and displayed a high Cd phenotype. Therefore, the phenotypic data categorized 137 individuals with a low Cd uptake level and six individuals with a high Cd uptake level (Table S6 and Table S7). Unfortunately, the DNA from three of these six individuals was not available because of a germination issue in the greenhouse. However, the genotypic data based on *Xusw47* and *IWB47298* showed that these germinated three individuals had alleles similar to the high Cd check, *i.e.*, the 220 bp amplicon for *Xusw47* and “C” SNP alleles for *IWB47298*. Comparatively, in 147 low Cd lines, we observed that almost half of the lines showed low Cd allele similar to Haurrani, and the remaining half of the lines showed low Cd alleles similar to D041735. As with the Strongfield × D041735 population, lines with the low Cd allele for *Xusw47* had the high Cd allele for *IWB47298*, and lines with the low Cd allele for *IWB47298* had the high Cd allele for *Xusw47*. The six high Cd lines had the high Cd uptake alleles for both markers.

The five and six high Cd RILs identified in the Strongfield × D041735 and D041735 × Haurani populations, respectively, represent recombination events and indicate that the two low Cd genes derived from D041735 and Strongfield/Haurani are different but closely linked. If low Cd uptake in D041735 was controlled by a different allele of the same gene as in Strongfield and Haurani, RILs with high levels of Cd would not be observed. It is interesting to note that we did not find any progenies with low Cd alleles from both sources. One possible explanation is that the presence of low Cd uptake alleles at both loci reduces plant viability, or is perhaps lethal. More work is needed to determine if the two alleles can be pyramided into a common genotype, and, if not, what biological mechanism(s) might prevent them from being able to exist in the same genome.

Strongfield is a Canadian cultivar with a pedigree of /Kyle/Niel/2/Ac Avonlea/DT665 (Clarke *et al.* 2005). The low Cd characteristic in Strongfield was obtained from Nile, which, like Haurani, is a landrace collected by the International Center of Agricultural Research in Dry Area, Syria, suggesting a possible common origin of low Cd gene. In contrast, D041735 is an experimental line developed from a cross between the experimental line D011543 and the cultivar Lebsock. D011543 was developed from a cross between the hexaploid wheat Sumai 3 and Lebsock. Because Sumai 3, like most hexaploid wheat cultivars, possesses the low Cd uptake characteristic, it is likely that it contributed the low Cd uptake trait to D041735. To test this, we genotyped D041735, Strongfield, Haurani, and Sumai 3 with the markers *Xusw47* and *IWB47298* (Figure 3). The results support the notion that D041735 obtained the marker alleles for these loci from Sumai 3. However, Wiebe *et al.* (2010) reported that the associated markers linked to *Cdu1-B* were not polymorphic in a population that was developed from a cross between Chinese Spring (CS-low Cd) and a CS-*Triticum dicoccoides* chromosome 5B disomic substitution line (CS-DIC 5B- high Cd). They hypothesized that it could be due to existence of another gene on the D-genome of hexaploid wheat controlling low Cd uptake in CS. However, the results of the current study suggest that the absence of marker polymorphism in the CS × CS-DIC 5B population, but presence of Cd uptake segregation, was probably due to that population segregating for the same gene that we identified in this research, that is the “hexaploid” source of low Cd uptake (Table 4). We suggest that using the KASP markers identified in this study on CS × CS-DIC 5B population probably would identify the linkage between these two genes.

**Table 4:** Summary of the genotyping results of previous and current markers linked to Cd uptake among three sources of grain low Cd level

Identified Markers for Cd Accumulation in Durum	Strongfield (Canadian Durum)	Haurrani (Land Race-Syria)	Allele for D041737 (NDSU Experimental Line)
Xusw47 (Wiebe 2012)	Low allele	Low allele	High allele
IWA1775 (AbuHammad *et al.* 2016)	Low allele	Low allele	Unknown
IWB47298 and IWB34332 (current research)	High allele	High allele	Low allele

Studies have shown significant variation in grain Cd content among modern durum wheat lines compared to hexaploid wheat, but Cd concentrations at the root surface in durum wheat and hexaploid wheat are not significantly different (Cakmak *et al.* 2000; Greger and Löfstedt 2004; Harris and Taylor 2001, 2004; Hart *et al.* 1998). However, there are many possible explanations, and, until the genes are both cloned and functionally characterized, a confident assumption cannot be made.

### Conclusion

Here, we studied the genetics of Cd uptake in the durum wheat line D041735 to identify markers for the selection of low Cd phenotype in breeding programs, and showed that D041735 carries a novel gene for Cd uptake resistance derived from hexaploid wheat. The close proximity of these two genes might suggest the presence of a gene hot spot in this region on the long arm of chromosome 5B in wheat that regulates heavy metal transporters. Markers *IWB55063* and *IWB47298* are highly effective in separating low Cd from high Cd uptake lines, and, thus, will be useful in speeding up the breeding progress for developing low Cd cultivars using marker-assisted selection.

We propose the symbol *Cdu2* to designate the low Cd uptake gene derived from D041735. In the future, map-based cloning of both *Cdu1-B* and *Cdu2-B* should provide insights into the evolutionary history of these genes and their mechanisms. As both sources are now available in durum wheat background, efforts to convert the repulsion phase of these two genes to coupling phase can be made to take full advantage of both low Cd uptake genes and additive genetic variation in durum wheat.

## Supplementary Material

Supplemental material is available online at www.g3journal.org/lookup/suppl/doi:10.1534/g3.117.300370/-/DC1.
