# Supplementary material for: Identification and Validation of a New Source of Low Grain Cadmium Accumulation in Durum Wheat
Source: G3 (Bethesda). 2018 Jan 19;8(3):923–32. doi: 10.1534/g3.117.300370 (PMC5844312; doi:10.1534/g3.117.300370)

Figure S1 Durum wheat linkage map generated from a cross between D041735 and Divide

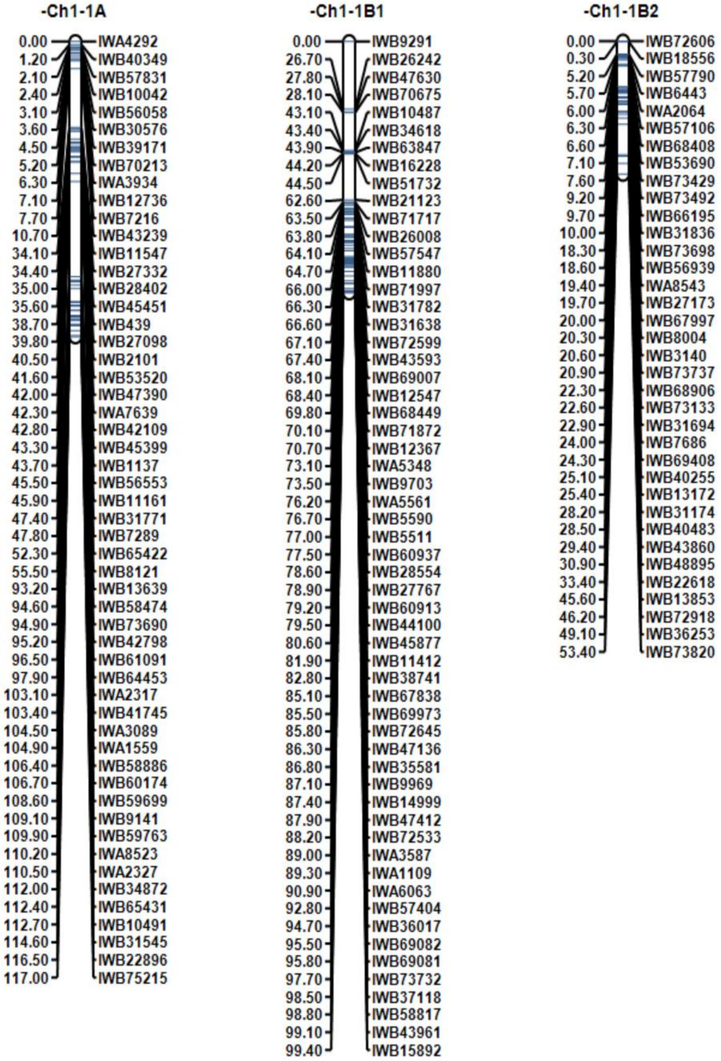

Figure S1 Durum wheat linkage map generated from a cross between D041735 and Divide

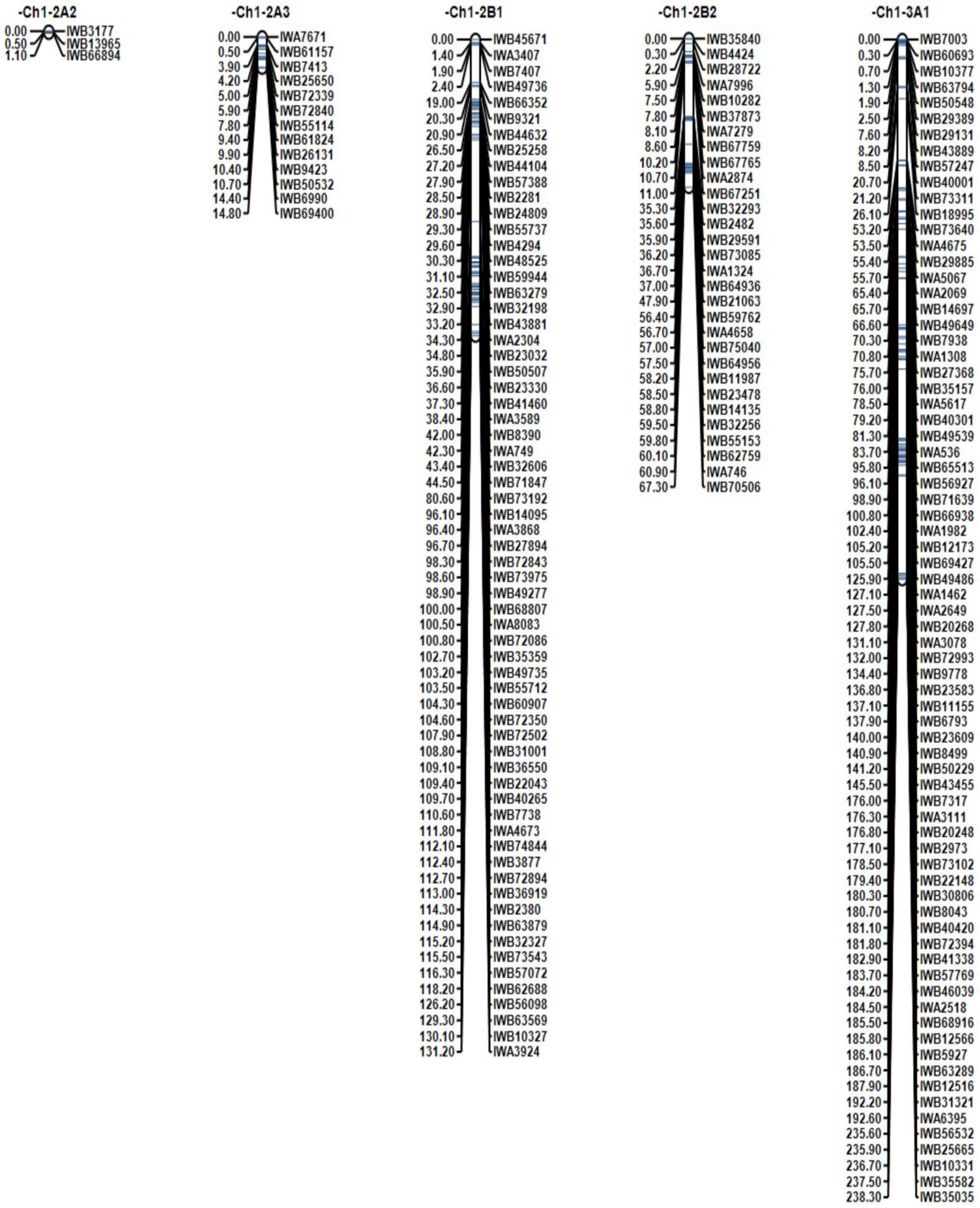

Figure S1 Durum wheat linkage map generated from a cross between D041735 and Divide

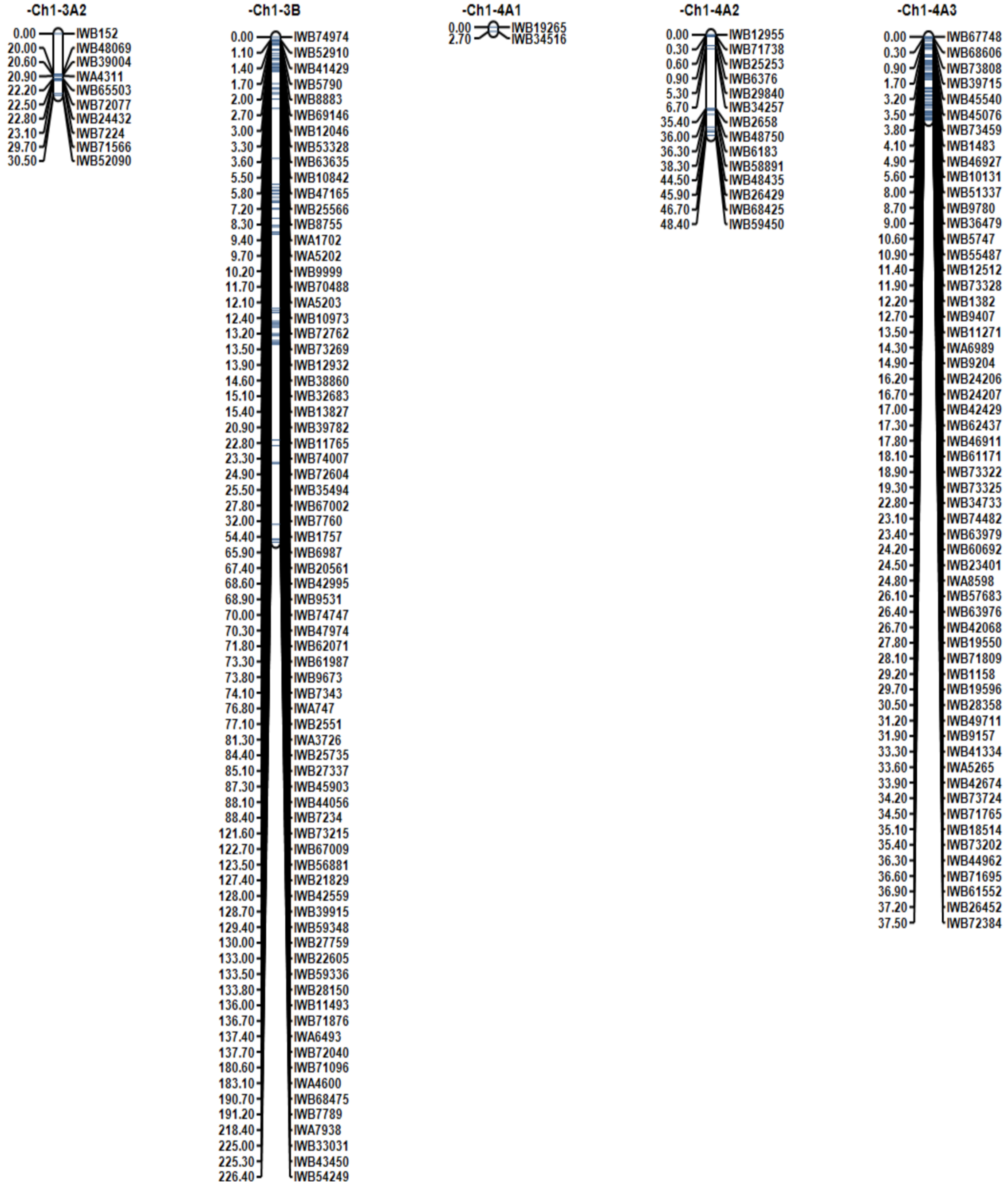

Figure S1 Durum wheat linkage map generated from a cross between D041735 and Divide

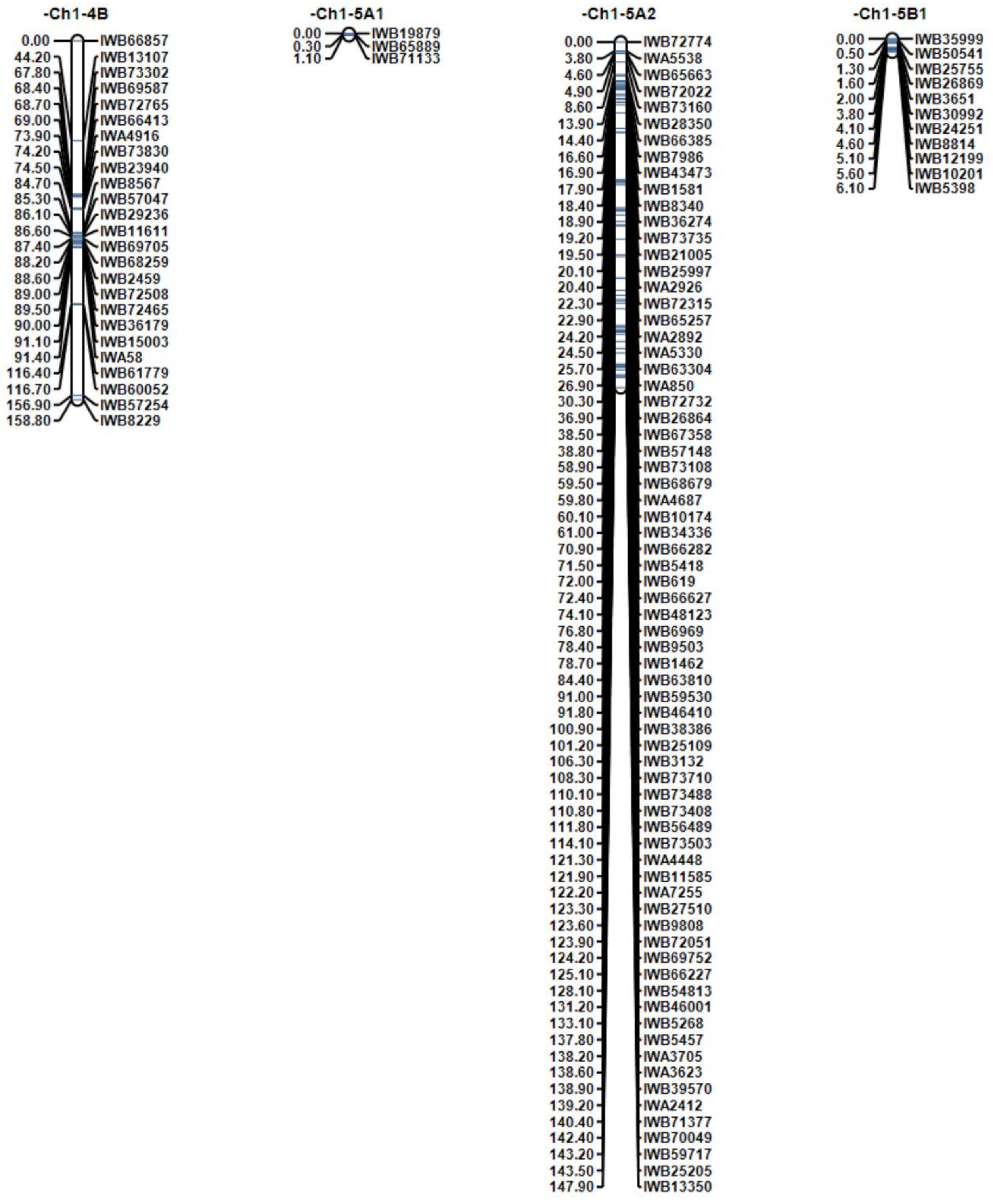

Figure S1 Durum wheat linkage map generated from a cross between D041735 and Divide

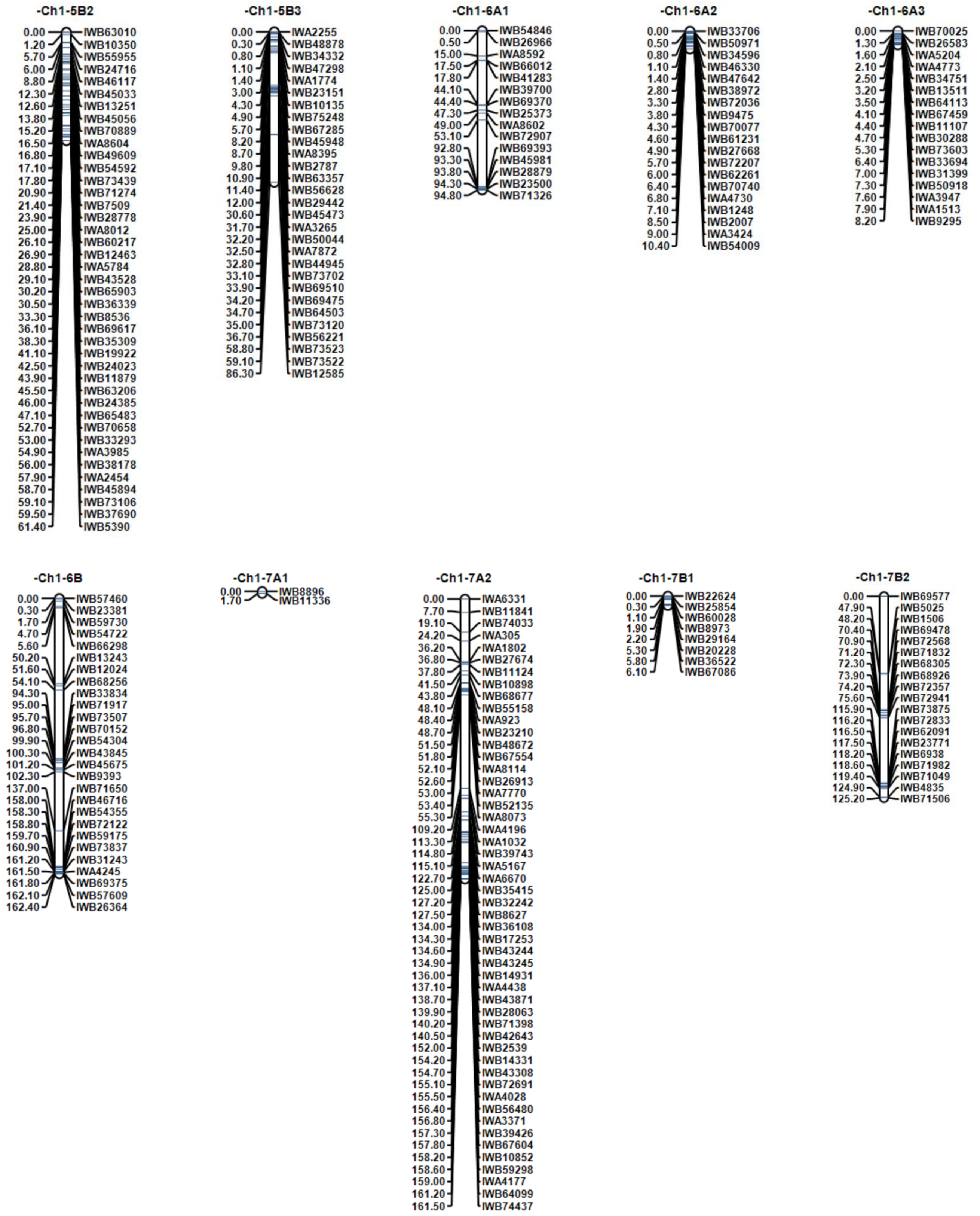

Supplement: Supplementary file 1 [file 923FigureS1.pdf]
