# Supplementary material for: Identification and Validation of a New Source of Low Grain Cadmium Accumulation in Durum Wheat
Source: G3 (Bethesda). 2018 Jan 19;8(3):923–32. doi: 10.1534/g3.117.300370 (PMC5844312; doi:10.1534/g3.117.300370)
Supplement: Supplementary file 5 [file 923TableS4.docx]

Table S4. Phenotypic performance of parents and checks in the D041735 × Strongfield RIL population

| D041735 × Strongfield (RIL population) | | |
| --- | --- | --- |
| **Parents** | Cd Content (mg/kg) | |
|  | Langdon | Prosper |
| D041735 | 0.016 | 0.221 |
| Strongfield | 0.025 | 0.240 |
| **Checks** |  | |
| Haurani | 0.017 | 0.235 |
| CD-Veronica | 0.046 | 0.267 |
| Carpio | 0.072 | 0.679 |
| Joppa | 0.047 | 0.469 |
| Divide | 0.066 | 0.430 |
| Tioga | 0.052 | 0.528 |
| **Parameter for Normal Distribution** |  | |
| Mean | 0.027 | 0.248 |
| Minimum | 0.014 | 0.144 |
| Maximum | 0.044 | 0.518 |
| Standard Deviation | 0.006 | 0.051 |
| LSD (0.05) | 0.011 | 0.101 |
