# Supplementary material for: Identification and Validation of a New Source of Low Grain Cadmium Accumulation in Durum Wheat
Source: G3 (Bethesda). 2018 Jan 19;8(3):923–32. doi: 10.1534/g3.117.300370 (PMC5844312; doi:10.1534/g3.117.300370)
Supplement: Supplementary file 6 [file 923TableS5.docx]

Table S5. Lines with a significantly high Cd phenotype derived from the cross D041735 × Strongfield

| D041735 × Strongfield (RIL population) | | |
| --- | --- | --- |
| Significantly high Cd lines | Cd Content (mg/kg) | |
|  | Langdon | Prosper |
| 83 | 0.032 | 0.391 |
| 89 | 0.031 | 0.518 |
| 91 | 0.042 | 0.423 |
| 110 | 0.028 | 0.360 |
| 133 | 0.036 | 0.362 |
