# Supplementary material for: Identification and Validation of a New Source of Low Grain Cadmium Accumulation in Durum Wheat
Source: G3 (Bethesda). 2018 Jan 19;8(3):923–32. doi: 10.1534/g3.117.300370 (PMC5844312; doi:10.1534/g3.117.300370)
Supplement: Supplementary file 7 [file 923TableS6.docx]

Table S6. Phenotypic performance of parents and checks in the mapping population D041735 × Haurani

| D041735 × Haurrani (RIL population) | | |
| --- | --- | --- |
| Parents | Cd Content (mg/kg) | |
|  | Langdon | Prosper |
| D041735 | 0.020 | 0.196 |
| Haurani | 0.022 | 0.258 |
| Checks |  | |
| Strongfield | 0.022 | 0.242 |
| CD-Veronica | 0.028 | 0.262 |
| Carpio | 0.127 | 0.778 |
| Joppa | 0.088 | 0.639 |
| Divide | 0.046 | 0.534 |
| Carpio | 0.121 | 0.786 |
| Parameter for Normal Distribution |  | |
| Mean | 0.028 | 0.260 |
| Minimum (no checks) | 0.005 | 0.117 |
| Maximum (no checks) | 0.080 | 0.592 |
| Standard Deviation | 0.010 | 0.069 |
| LSD (0.05) | 0.021 | 0.137 |
