# Supplementary material for: Identification and Validation of a New Source of Low Grain Cadmium Accumulation in Durum Wheat
Source: G3 (Bethesda). 2018 Jan 19;8(3):923–32. doi: 10.1534/g3.117.300370 (PMC5844312; doi:10.1534/g3.117.300370)
Supplement: Supplementary file 8 [file 923TableS7.docx]

Table S7. Lines with a significantly high Cd phenotype derived from the cross D041735 × Haurani

| D041735 × Haurrani (RIL population) | | |
| --- | --- | --- |
| Significantly high Cd lines | Cd Content (mg/kg) | |
|  | Langdon | Prosper |
| 48 | 0.080 | 0.541 |
| 63 | 0.066 | 0.592 |
| 126 | 0.055 | 0.405 |
| 127 | 0.053 | 0.527 |
| 135 | 0.046 | 0.408 |
| 139 | 0.043 | 0.402 |
